# Study of age-related changes in plasma metabolites and enzyme activity of healthy small dogs that underwent medical checkups

**DOI:** 10.3389/fvets.2024.1437805

**Published:** 2024-10-24

**Authors:** Akio Kusaba, Erika Tago, Haruna Kusaba, Koh Kawasumi

**Affiliations:** ^1^Laboratory of Veterinary Biochemistry, Nippon Veterinary and Life Science University, Musashino, Japan; ^2^Muromi Animal Hospital, Fukuoka, Japan

**Keywords:** adiponectin, age-related inflammation, healthy dog, medical checkup, SAA

## Abstract

**Introduction:**

In Japan, the importance of medical checkups for pet dogs is increasing. In this study, we retrospectively explored the effects of age on plasma biomarkers in healthy small dogs that underwent medical checkups.

**Methods:**

Based on the modified American Animal Hospital Association Canine Life Stage Guidelines, 52 healthy small dogs were divided into 3 groups according to their life stage: young adult (1–4 years old), mature adult (5–11 years old), senior (12–15 years old). None of the dogs were obese. Plasma was collected from animals that underwent medical checkups at Muromi Animal Hospital (Fukuoka, Japan). Plasma glucose, triglyceride (TG), total protein, blood urea nitrogen (BUN), creatinine, total cholesterol, and albumin concentrations; alanine aminotransferase, aspartate aminotransferase, and alkaline phosphatase (ALP) activities; c-reactive protein (CRP), non-esterified fatty acid, malondialdehyde (MDA), serum amyloid A (SAA), insulin, and adiponectin (ADN) concentrations; glutathione peroxidase (GPx), superoxide dismutase (SOD), malate dehydrogenase (MDH), and lactate dehydrogenase (LDH) activities; and M/L ratio (MDH/LDH) were examined. Changes in the abovementioned plasma biomarker levels were compared between canines in different life stages.

**Results:**

Plasma ADN concentrations and GPx, SOD, and MDH activities significantly decreased with age, whereas plasma ALP, BUN, TG, and MDA concentrations gradually increased. Plasma SAA levels measured by the latex agglutination method in 51 of the 52 small dogs that underwent medical checkups were below the detection limit.

**Conclusion:**

Plasma ADN concentrations, GPx, SOD activity, and BUN levels may be important biomarkers for clarifying the effect of age in healthy dogs that undergo medical checkups. However, plasma SAA values obtained by the latex agglutination method were not considered an age-related inflammation marker for healthy dogs.

## 1 Introduction

In the field of veterinary medicine, as in human medicine, there is a growing movement to promote “prevention” and “prevention of disease.” There is a particularly growing awareness of the importance of aging-related care in pets (1–6), as aged dogs are likely to become obese and develop other metabolic diseases that induce chronic inflammation. Senescent cells secrete various factors such as inflammatory cytokines, chemokines, and extracellular matrix-degrading enzymes that have inflammatory and carcinogenic effects, causing a phenomenon called the senescence-associated secretory phenotype (7–10), which is associated with a variety of diseases (11).

The emphasize on prevention of disease increased the need for medical checkups for pets, as regular medical checkup programs for laboratory dogs are essential for their well-being (12). In Japan, veterinary medical checkups for pets include general inspection tests such as 15–20 blood tests, urinalysis, stool tests, and definitive diagnostic tests, such as chest/abdominal x-ray examinations, abdominal ultrasound tests, electrocardiograms, blood pressure measurements, and thyroid hormone tests. A medical checkup is a detailed examination to detect diseases that may be overlooked during an annual health checkup performed at the time of rabies vaccination. However, even in animals that undergo annual medical checkups and are diagnosed as healthy, illnesses can be discovered before 1 year. Moreover, almost all tests that can be performed without anesthesia are performed at primary care facilities. Therefore, it is necessary to consider whether there are any items that can detect metabolic changes earlier by measuring additional aging and metabolic biomarkers that may not be routinely assessed in the hospital.

Franceschi et al. (13) proposed the term “inflammaging,” which refers to a proinflammatory state associated with the low-grade inflammation associated with aging. Several researchers in veterinary medicine have also reported inflammaging (14–21). Levels of acute phase protein, an acute-phase inflammatory marker, has also been used to investigate chronic inflammatory conditions. Particularly, serum amyloid A protein (SAA) levels increase with age in various diseases. Currently, SAA values are considered disease onset markers (22, 23). SAA is thought to be a major contributor to the obesity-associated inflammatory response (24). SAA promotes the lipolysis of adipose tissue through the NF-κB system, increases blood free fatty acid concentration, and induces insulin resistance and vascular damage (25). However, it remains unclear whether increased plasma SAA levels are caused by obesity-induced or age-related inflammation (inflammaging) in aged obese dogs.

Therefore, in this study, we examined the changes in plasma biomarker values such as plasma metabolites, hormone concentrations, and enzyme activities in healthy dogs without obesity that underwent medical checkups, and life stage differences were investigated. In addition, we investigated whether plasma SAA levels measured by the latex agglutination method could be used as an indicator of age-related inflammation in healthy aging dogs. Notably, because the lifespan of dogs varies depending on size—small dogs tend to live longer than larger animals (26)—and the age of small dogs corresponding to humans is less than that of larger dogs (27, 28), the effect of age on metabolism should be evaluated using a uniform size. Therefore, because small dogs account for the largest population in our country (29), we focused on small healthy dogs that underwent medical checkups to detect age-related inflammation.

## 2 Materials and methods

### 2.1 Animals

Fifty-two clinically healthy small dogs that underwent medical checkups at the Muromi Animal Hospital (Fukuoka City) were recruited. Based on the modified AAHA Canine Life Stage Guidelines (30), the animals were divided into three groups: young adult (1–4 years old), mature adult (5–11 years old), senior (12–15 years old).

Ethical approval was obtained from Muromi Animal Hospital (R23-1). Written informed consent was obtained from each owner. The frozen plasma samples collected during the medical checkups were taken to Nippon Veterinary and Life Science University, and plasma biomarkers were examined.

### 2.2 Body type classification

Considering the species of dogs in this study, those weighing less than 9 kg were regarded as small dogs (28).

### 2.3 Body weight (BW) and body conditioning score (BCS) measurement

BW was measured during the medical checkups. To evaluate the BCS of the dogs, we employed a 9-point system: 1/9, emaciated; 2/9, very thin; 3/9, thin; 4/9, underweight; 5/9, ideal; 6/9, overweight; 7/9, heavy; 8/9, obese; and 9/9, severely obese (31).

### 2.4 Blood sampling and analysis

Fasting blood was collected from the lateral saphenous vein into heparinized tubes after at least 8 h of fasting. Plasma was collected by centrifugation at 3500G for 5 min at room temperature and stored at −80°C until use. Glucose, triglyceride (TG), total protein (TP), blood urea nitrogen (BUN), creatinine (CRE), total bilirubin, total cholesterol (TC), albumin (ALB) concentrations and alanine aminotransferase, aspartate aminotransferase (AST), alkaline phosphatase (ALP) activities, C-reactive protein (CRP) were measured at Muromi Animal Hospital (Fukuoka, Japan) using an automatic biochemical analyzer, FUJI dry chem 7,000 V (Fujifilm Medical Co., Ltd., Tokyo, Japan).

Using the remaining plasma samples collected for medical checkups, non-esterified fatty acids (NEFA), malondialdehyde (MDA), serum amyloid A protein (SAA), insulin, adiponectin (ADN) concentrations, glutathione peroxidase (GPx), superoxide dismutase (SOD), malate dehydrogenase (MDH), lactate dehydrogenase (LDH) activity, and M/L ratio (MDH/LDH) were measured retrospectively at the Veterinary Biochemistry Laboratory, Nippon Veterinary and Life Science University (Tokyo, Japan). NEFA levels were measured using a commercial kit (NEFA C-Test Wako; Fujifilm Wako Pure Chemical Industries, Ltd. Tokyo, Japan). MDA was measured using a commercial kit (NWLSSTM Malondialdehyde Assay Kit, NWLSS [#NWK-MDA01], Northwest Life Science Specialties, LLC, Vancouver, Canada). Insulin was measured using a commercial kit (REVIS® Insulin-Rat T, Fujifilm Wako Shibayagi Co., Ltd., Gunma, Japan). ADN levels were measured using a commercial kit (mouse/rat adiponectin ELISA kit; Otsuka Pharmaceutical Co., Ltd., Tokyo, Japan). Fifty-thousand-fold diluted plasma samples were used for ADN measurement. The acceptable detection limit was estimated to be from 0.25 ng/mL to 8.0 ng/mL by calibration curve (32). GPx levels were measured using a commercial kit (NWLSS Glutathione Peroxidase Assay Kit; Northwest Life Science Specialties, LLC, Vancouver, Canada). SOD activity was measured using a commercial kit (Superoxide Dismutase ELISA Kit, Northwest Life Science Specialties, LLC, Vancouver, Canada). LDH and MDH activities were measured using the methods reported by Kaloustian et al. (1969) (33) and Bergmeyer and Bernt (1974) (34), respectively. LDH activity was calculated by measuring the change in absorbance at 340 nm for 180 s using an absorbance meter while MDH activity was calculated by measuring the change in absorbance at 340 nm for 300 s using an absorbance meter. The M/L ratio was calculated by dividing the MDH level by the LDH level.

Plasma SAA values were measured at the Research and Development Department of Eiken Chemical Co., Ltd. (Tochigi, Japan) using a Hitachi 7,180 automatic analyzer with the measurement reagent VET-SAA (Eiken Chemical, Tokyo, Japan). Age-related changes in the abovementioned plasma biomarkers were compared according to canine life stages.

### 2.5 Statistical analysis

Results are presented as mean ± standard error (SE). Statistical significance was determined using one-way analysis of variance (ANOVA). The significance level was set at *p* < 0.05 or *p* < 0.01. Correlation coefficients and *p*-values between age and the examined parameters were calculated using multivariate regression analysis. Significance levels were set at *p* < 0.05 or *p* < 0.01. Correlation coefficients were evaluated as follows: |0.7–1.0|: excellent, |0.4–0.7|: moderate, |0.2–0.4|: weak, |0–0.2|: no correlation.

## 3 Results

As shown in Table 1, the 52 healthy small dogs that underwent medical checkups were divided into 3 groups according to their life stage: 6 young adults, 35 mature adults, and 11 seniors. The mean ± SE of body weight for the 52 small dogs was 5.68 ± 0.30 kg. Among the 52 dogs, there were 3 Miniature Schnauzers, 2 Miniature Dachshunds, 19 Toy Poodles, 1 West Highland White Terrier, 2 Cavaliers, 5 Shizu dogs, 2 Shiba dogs, 3 Chihuahuas, 2 Pugs, 3 Pekingese dogs, 3 Pomeranians, 1 Maltese, 3 Yorkshire Terriers, and 3 mixed breeds. Fifty-one of the 52 small dogs had a BCS of 5 on a 9-point scale and were not obese.

**Table 1:** Changes in BW and BCS in healthy small dogs examined in medical checkups.

		Young adult (6)			Mature adult (35)			Senior (11)		
Age	(Years)	3.9	±	0.6	9.0	±	0.3	14.0	±	0.4
Weight	(kg)	6.6	±	1.1	5.8	±	0.4	4.7	±	0.5
BCS	(/9)	5.5	±	0.3	5.5	±	0.2	5.0	±	0.1

Data are presented as mean ± SE.

The numbers in parentheses indicate the number of animal examined.

BW, body weight; BCS, body condition score; SE, standard error.

Although statistical significance was not observed, plasma BUN, TG, and MDA levels tended to gradually increase with age, whereas plasma ADN concentrations in the senior group were significantly decreased compared with those of young adult group and mature adult group (one-way ANOVA, *p* = 0.0048, *p* = 0.0241, respectively: Table 2). Although the difference was not significant, plasma TP levels tended to increase with age, whereas plasma ALB levels tended to decrease with age.

**Table 2:** Changes in plasma metabolites and hormone concentrations in healthy small dogs examined in medical checkups.

		Young adult (6)			Mature adult (35)			Senior (11)		
GLU	(mg 100 mL^−1^)	106.7	±	7.5	102.7	±	1.4	104.0	±	2.4
TG	(mg 100 mL^−1^)	61.7	±	6.7	94.6	±	15.3	157.6	±	50.0
TC	(mg 100 mL^−1^)	223.7	±	39.6	217.8	±	14.0	227.6	±	18.8
NEFA	(mEq L^−1^)	1.25	±	0.3	1.18	±	0.10	1.05	±	0.17
TP	(g 100 mL^−1^)	6.3	±	0.2	6.4	±	0.1	6.6	±	0.3
ALB	(g 100 mL^−1^)	3.5	±	0.2	3.4	±	0.1	3.3	±	0.1
CRE	(mg 100 mL^−1^)	0.7	±	0.1	0.7	±	0.0	0.9	±	0.1
BUN	(mg 100 mL^−1^)	16.1	±	1.5	16.3	±	1.3	34.3	±	6.3
MDA	(μmol L^−1^)	0.7	±	0.4	0.9	±	0.3	1.1	±	0.4
CRP	(mg 100 mL^−1^)		NT		0.8	±	0.2	0.9	±	0.2
INS	(ng mL^−1^)	0.2	±	0.1	0.4	±	0.2	0.2	±	0.1
ADN	(μmol mL^−1^)	40.4	±	10.3*	26.8	±	3.3*	12.5	±	2.3

Data are presented as mean ± SE.

The numbers in parentheses indicate the number of animals examined.

* Significant (*p* < 0.05) compared to the senior group (one-way ANOVA).

ADN, adiponectin; ALB, albumin; ANOVA, analysis of variance; BUN, blood urea nitrogen; CRP, c-reactive protein; CRE, creatinine; GLU, glucose; INS, insulin; MDA, malondialdehyde; NEFA, non-esterified fatty acid; SE, standard error; TC, total cholesterol; TG, triglyceride; TP, total protein.

The GPx and MDH activities in the senior group were significantly lower than those in the mature group (one-way ANOVA, *p* = 0.0049 and *p* = 0.0182, respectively; Table 3). Plasma SOD activity in the mature adult group was significantly lower than that in the young adult group (*p* = 0.044).

**Table 3:** Changes in plasma enzymes activities in healthy small dogs examined in medical checkups.

		Young adult (6)			Mature adult (35)			Senior (11)		
AST	(U/L)	31.2	±	3.9	33.2	±	2.5	28.1	±	2.2
ALT	(U/L)	44.3	±	4.4	77.2	±	8.6	86.5	±	23.1
ALP	(U/L)	95.4	±	28.8	170.3	±	39.4	325.9	±	79.4
GPx	(mU/mL)	26.0	±	4.3	24.3	±	1.3	18.7	±	2.8**
SOD	(unit/mL)	44.7	±	23.4**	19.3	±	3.3	11.0	±	1.7
MDH	(IU/L)	59.8	±	19.5	73.1	±	6.2	44.6	±	6.0**
LDH	(IU/L)	88.0	±	24.3	109.0	±	9.6	91.6	±	12.6
M/L		0.6	±	0.1	0.8	±	0.2	0.5	±	0.1

The data are expressed as mean ± SE.

The numbers in parentheses indicate the number of animals examined.

** Significant (*p* < 0.05) when compared against mature adult group (One-way ANOVA).

ALP, alkaline phosphatase; ALT, alanine aminotransferase; ANOVA, analysis of variance; AST, aspartate aminotransferase; GPx, glutathione peroxidase; LDH, lactate dehydrogenase; MDH, malate dehydrogenase; M/L, malate dehydrogenase/lactate dehydrogenase ratio; SE, standard error; SOD, superoxide dismutase.

As shown in Table 4, the plasma SAA levels in 51 of the 52 healthy small dogs that underwent medical checkups were below the lower detection limit, which was calculated as 3.54 mg/L (35). One dog without severe inflammation (age: 9.5 years, BCS: 6/9; with cataracts, pupillary atrophy, and mild periodontal disease) had a plasma SAA value of 12.3 mg/L.

**Table 4:** Plasma SAA levels in 52 small dogs.

	Young adult (6)				Mature adult (35)						Senior (11)			
Age (dog)	1	3	4	5	6	7	8	9	10	11	12	13	14	15
(Human)	15	28	32	36	40	44	48	52	56	60	64	68	72	76
SAA (mg L^−1^)	0.6	0.6	0.4	0.8	0.7	0.6	0.5	0.7	0.4	1.0	0.9	1.2	0.5	0.7
			0.5	0.8	0.6	0.7	0.6	0.8	0.3	0.6	1.0	0.9	1.7	2.0
			0.9		0.3	0.1	0.8	0.3	0.6	0.8	1.3	0.2		0.8
			0.6		0.6	0.5	0.8	0.5	0.9	0.5				
						2.0		0.5		0.0				
								12.3		0.8				
								1.1						
								0.3						
								0.7						
								0.3						
AV	0.6	0.6	0.6	0.8	0.6	0.8	0.7	1.8	0.6	0.6	1.1	0.8	1.1	1.2

SAA, serum amyloid A.

The correlation coefficients and *p*-values between age and biomarker levels were calculated using multivariable regression analysis (Table 5). The following markers were positively correlated with age: BUN levels, moderately positive (coefficient 0.499, *p* = 0.000); CRE levels, moderately positive (coefficient 0.467, *p* = 0.001); TG levels, weakly positive (coefficient 0.237, *p* = 0.138); ALP levels, weakly positive (coefficient 0.297, *p* = 0.024); and TP levels, weakly positive (coefficient 0.204, *p* = 0.246). Negative correlations were as follows: ADN levels, moderately negative (coefficient − 0.478, *p* = 0.007); GPx activity, moderately negative (coefficient − 0.414, p = 0.007); SOD activity, weakly negative (coefficient − 0.267, *p* = 0.127); and AST levels, weakly negative (coefficient − 0.242, *p* = 0.132). Plasma MDH activity (an energy metabolism marker) and plasma SAA level (an inflammatory marker) did not correlate with age (coefficient − 0.144, p = 0.007; coefficient 0.065, *p* = 0.683, respectively).

**Table 5:** Correlation coefficients and *P* values between the age and examined parameters in 52 healthy dogs.

	Correlation coefficient	*P* value
BW	−0.198	0.105
BCS	−0.168	0.150
GLU	−0.015	0.713
TC	0.044	0.262
TG	0.237	0.138
NEFA	−0.025	0.837
MDA	0.129	0.482
AST	−0.242	0.132
ALT	0.133	0.379
ALP	0.297	0.024
MDH	−0.144	0.007
LDH	0.015	0.727
M/L ratio	−0.087	0.439
SOD	−0.267	0.127
GPx	−0.414	0.007
ADN	−0.478	0.007
INS	0.004	0.983
TP	0.204	0.246
ALB	−0.033	0.419
BUN	0.499	0.000
CRE	0.467	0.001
SAA	0.065	0.683

Correlation coefficients were evaluated as follows: |0.7–1.0|: excellent, |0.4–0.7|: moderate, |0.2–0.4|: weak, |0–0.2|: no correlation using Pearson analysis. P-values were evaluated using multivariable regression analysis.|Correlation coefficients were evaluated as follows: |0.7–1.0|: excellent, |0.4–0.7|: moderate, |0.2–0.4|: weak, |0–0.2|: no correlation using Pearson analysis. P-values were evaluated using multivariable regression analysis.|Correlation coefficients were evaluated as follows: |0.7–1.0|: excellent, |0.4–0.7|: moderate, |0.2–0.4|: weak, |0–0.2|: no correlation using Pearson analysis. P-values were evaluated using multivariable regression analysis.|Correlation coefficients were evaluated as follows: |0.7–1.0|: excellent, |0.4–0.7|: moderate, |0.2–0.4|: weak, |0–0.2|: no correlation using Pearson analysis. P-values were evaluated using multivariable regression analysis.|Correlation coefficients were evaluated as follows: |0.7–1.0|: excellent, |0.4–0.7|: moderate, |0.2–0.4|: weak, |0–0.2|: no correlation using Pearson analysis. P-values were evaluated using multivariable regression analysis.|Correlation coefficients were evaluated as follows: |0.7–1.0|: excellent, |0.4–0.7|: moderate, |0.2–0.4|: weak, |0–0.2|: no correlation using Pearson analysis. P-values were evaluated using multivariable regression analysis.|Correlation coefficients were evaluated as follows: |0.7–1.0|: excellent, |0.4–0.7|: moderate, |0.2–0.4|: weak, |0–0.2|: no correlation using Pearson analysis. P-values were evaluated using multivariable regression analysis.|Correlation coefficients were evaluated as follows: |0.7–1.0|: excellent, |0.4–0.7|: moderate, |0.2–0.4|: weak, |0–0.2|: no correlation using Pearson analysis. P-values were evaluated using multivariable regression analysis.|Correlation coefficients were evaluated as follows: |0.7–1.0|: excellent, |0.4–0.7|: moderate, |0.2–0.4|: weak, |0–0.2|: no correlation using Pearson analysis. P-values were evaluated using multivariable regression analysis.

ADN, adiponectin; ALB, albumin; ALP, alkaline phosphatase; ALT, alanine aminotransferase; AST, aspartate aminotransferase; BCS, body condition score; BUN, blood urea nitrogen; BW, body weight; CRE, creatinine; GLU, glucose; GPx, glutathione peroxidase; INS, insulin; LDH, lactate dehydrogenase; MDA, malondialdehyde; M/L, malate dehydrogenase/lactate dehydrogenase ratio; NEFA, non-esterified fatty acid; SAA, serum amyloid A; SOD, superoxide dismutase; TC, total cholesterol; TG, triglyceride.

## 4 Discussion

The characteristics of medical checkups in Japan are different from those of health checkups conducted once a year. During medical checkups, more detailed examinations are performed based on the wishes of pet owners concerned about the onset of potential diseases. Therefore, even in pets that are diagnosed as healthy during health checkups, we check for the presence or absence of various internal abnormalities in the body, such as latent inflammatory diseases and tumors, as early as possible.

Since small dogs are more commonly kept compared to medium and large dogs in Japan, we focused on small dogs in this study. The equivalent age at each life stage differs between small, medium, and large dogs, and it is impossible to evaluate the effects of age on metabolic changes in the same way.

### 4.1 Changes in plasma enzyme activities

GPx is an enzyme belonging to the selenoprotein family that catalyzes the reduction of hydrogen peroxide and lipid peroxides (36). Four types of GPx (GPx-1–4) have been identified in mammals (37). GPx4 is an antioxidant enzyme known to directly reduce phospholipid hydroperoxides in membranes and lipoproteins and cooperates with *α*-tocopherol to suppress lipid peroxidation (38). In this study, plasma GPx and SOD activities tended to decrease in aged dogs that underwent medical checkups. This suggests that the protective function against cytotoxicity owing to the strong oxidizing action of superoxide may weaken with age.

### 4.2 MDH, M/L ratio

MDH (L-malate: NAD oxidoreductase [EC 1.1.1.37]) catalyzes the NAD/NADH-dependent interconversion of the substrates, malate and oxaloacetate. This reaction plays an important role in the malate/aspartate shuttle across the mitochondrial membrane and the tricarboxylic acid cycle within the mitochondrial matrix (39).

In this study, MDH activity significantly decreased at the senior stage compared to that at the mature adult stage. This suggests a decrease in energy metabolism in the senior stage, as has been observed in humans. The M/L ratio, calculated by dividing the activity value by the LDH activity value, is thought to be an index for evaluating the metabolic state of animal tissues (40). It is used as a sensitive marker of increased energy metabolism, including increased ATP production, in tissues (41). Previous research on the relationship between aging and the M/L ratio in riding horses also found no significant difference in the M/L ratio between young and old groups (42). To investigate age-related energy metabolism, MDH activity may be more appropriate than the M/L ratio.

### 4.3 ALP

ALP [orthophosphoric-monoester phosphorylase (EС 3.1.3.1)] is an enzyme with low substrate specificity that hydrolyzes phosphate monoester bonds under alkaline conditions (pH 9–11). In clinical practice in dogs, it is primarily used as an indicator of liver and biliary tract diseases (43). In dog serum, three ALP isoenzymes are known: bone ALP, liver ALP (LALP), and corticosteroid-induced ALP (44). LALP accounted for less than 10% of young dogs, but more than 50% of total ALP in middle-aged and elderly dogs (45). In this study, the age-related increase in plasma ALP levels was suggested to be due to an increase in LALP levels.

### 4.4 Changes in plasma metabolites

Plasma TG levels tended to increase with age. This indicates that lipid metabolism tends to decline with age, even if it does not manifest as an abnormality.

Due to decreased lipid metabolism, fat accumulates in areas other than the visceral fat and adipose tissue, leading to obesity, and the ectopic accumulation of fat in the muscle and liver induces inflammation (46). In addition, obesity activates aging signals in adipose tissue and promotes immunosenescence (47). However, in this study, not all dogs that underwent pet medical checkups displayed obesity-related inflammation, considering their BCS and plasma SAA levels.

An increase in plasma TP levels and decrease in plasma ALB levels indicate an increase in plasma globulin levels. In such cases, chronic inflammation such as periodontal disease is often observed.

Plasma BUN values are influenced by three factors: ingested proteins and protein catabolism, urea synthesis by the liver and excretion by the kidneys, and changes depending on the amount of urea filtered glomerular filtration. Therefore, it is considered less reliable than CRE levels (48). However, an increase in plasma BUN levels accompanied by an increase in plasma CRE levels in the senior stage may be an important indicator of kidney disease. Consequently, urine specific gravity, urine protein, and blood pressure measurements should be considered. In obese cats, serum BUN levels significantly increased during the geriatric stage (49).

MDA is a relatively stable end product of the peroxidation of polyunsaturated fatty acids and is considered an indicator of oxidative stress (50, 51). The measurement results showed a tendency for plasma MDA levels to increase in dogs with age, although the difference was not significant. We hypothesized that oxidative stress may increase in aged dogs undergoing medical checkups. In addition, the levels of GPx and SOD, which have antioxidant effects, significantly decrease with age. These antioxidant effects were consistent with an increase in plasma MDA levels. Notably, many aging phenomena are suggested to be related to oxidative stress. In dogs and cats, it is said to be related to degenerative changes in the renal tubules (52). It was speculated that the increase in BUN and CRE is due to oxidative stress, which leads to early kidney disease. Previously, we attributed elevated BUN levels to increased protein intake in aging patients. By measuring GPx, SOD, and MDA, we may be able to differentiate between oxidative stress and excessive protein intake as potential causes.

Plasma ADN concentrations showed the highest levels (40.4 ± 10.3 μg/mL) at the young adult stage. Subsequently, the concentrations decreased with age. This suggests that the anti-inflammatory effect might decrease, and insulin resistance might increase in healthy aged dogs who undergo medical checkups, even if they are not obese. In a previous study, we examined plasma ADN concentrations in experimentally induced healthy obese dogs fed a high-fat diet. ADN concentrations at the young adult life stages were at the same level as in this study (38.8 ± 7.8 μg/mL) (53). In addition, the positive correlation of TG and ALP with age suggests that decreased ADN activity may lead to reduced fat burning and bile stagnation in the liver and elevated TG levels due to decreased AMP kinase activation, which increases ALP levels. Moreover, serum ALP activity increases in dogs and cats are reportedly due to intrahepatic or extrahepatic cholestasis (43). Since the owners of animals undergoing medical checkups are very health-conscious, they may not be obese according to BCS, but they may have accumulated visceral fat. In Japan, many owners feed their dogs jerky as a treat, and many of these snacks contain artificial additives. If a small dog continues to ingest additives every day, it puts more strain on the liver and liver enzymes rise. In cases of high TG and TC levels or cases of elevated liver enzymes without abnormalities in the liver parenchyma on ultrasonography, we have prescribed dietary foods (high fiber foods) or low fat foods. However, if we can confirm a decrease in ADN in our hospital, supplementing with 5-aminolevulinic acid might be beneficial, as it has been shown to reduce TG levels and liver enzymes (54, 55).

### 4.5 SAA

At the beginning of this study, we hypothesized that plasma SAA levels, measured using Vet SAA (Eiken Chemical), could serve as an indicator of age-related inflammation. A paper comparing apolipoprotein-A1 in septic shock and multiple organ dysfunction syndrome using Eiken Chemical’s Vet SAA (the same one we used) reported that both CRP and SAA showed high values (56). This paper reported that the average values of SAA in 21 cases of septic shock were 400 μg/mL and CRP values were 23.6 mg/dL. We expected that Vet SAA (Eiken Chemical) would have a higher value and wider detection range during the same inflammation compared to CRP, an apolipoprotein-A1 commonly used in dogs.

As mentioned above, it is assumed that slight inflammation occurs in the animals’ bodies due to oxidative stress caused by aging, and it was hypothesized that the onset of this very small inflammation could be detected using Vet SAA, which would result in differences between groups. In addition, the young adult group is excluded from the test items in our hospital’s medical checkups because they are young and often do not have inflammation, but the CRP levels measured in the mature adult and senior groups were low at 0.8 ± 0.2 and 0.9 ± 0.2 mg/dL, respectively, but were above the reference range. Therefore, some kind of inflammation may have begun to occur in the body. However, almost all the plasma SAA levels were below the detection limit. Therefore, although it has been confirmed that plasma SAA levels measured by the latex agglutination method are elevated in cases of severe inflammatory diseases, it may be difficult to detect the onset of inflammation that we are looking for using SAA. Furthermore, to apply other detection methods such as ELISA and chemiluminescence, it is necessary to verify plasma SAA levels as a marker of age-related inflammation.

Dogs are important for the investigation of age-related diseases in humans (57). Like humans, elderly dogs tend to become obese (58). In aged obese dogs, it is difficult to determine whether inflammation is induced by aging or obesity. To verify the onset of inflammation due to aging, we measured the degree of inflammation in clinically healthy dogs without obesity, by measuring plasma SAA levels using the latex agglutination method. As a result, the plasma SAA level was below the detection limit, and no pathological inflammation related to aging was observed. Future studies should investigate whether the latex agglutination test for SAA remains below the detection limit in obese senior dogs. Notably, only one of the 52 dogs in this study had a measurable SAA level, and this dog had a BCS of 6/9. Healthy dogs that have undergone pet checkups do not show the pathological inflammation associated with aging. Pet owners are afraid of latent inflammatory illnesses that cannot be identified through health checkups. In this study, the plasma SAA levels in healthy dogs provided sufficient information to pet owners.

### 4.6 Limitations

None of the small dogs recruited in this study were obese, and they had no clinical symptoms, although Miniature Schnauzers have breed-specific altered TG metabolism (59, 60) and are likely to become hyperlipidemic with age (61). Therefore, we do not believe that these results are representative of all small dogs bred in Japan. However, our results regarding the characteristics on age-related changes in plasma enzyme activities, hormones, and metabolites may be a characteristic of healthy small dogs that have undergone medical checkups.

Although we targeted small dogs that underwent medical checkups in this study, small dogs that undergo regular health examinations may have different physiological characteristics, such as obesity. Therefore, it is necessary to conduct further studies on dogs from other populations to clarify the effects of age. Since medium-sized and large dogs have different lifespans than small dogs, they are thought to have different age-related metabolic characteristics. Therefore, we need to verify the characteristics of age-related inflammation separately. Plasma SAA measurements using latex agglutination tests can also be performed in dogs with inflammatory diseases. Therefore, it may be difficult to detect early-stage age-related inflammation in non-obese small dogs.

## 5 Conclusion

In this study, age-related changes in plasma BUN levels, ADN concentrations, and GPx, SOD and MDH activities were found in healthy small dogs that underwent medical checkups. These may complement in-hospital testing and contribute to earlier detection of disease. Further studies on more biomarkers are required to better understand age-related inflammation. We measured SAA using the latex agglutination method to determine whether it could be used as an indicator of age-related inflammation. However, this test was unable to detect age-related inflammation in dogs without inflammatory diseases because most of the clinically healthy canine samples were below the lower detection limits. We confirmed that the healthy dogs that underwent medical checkups had no inflammation in their inner bodies.

## Data Availability

The original contributions presented in the study are included in the article/supplementary material, further inquiries can be directed to the corresponding author/s.

## Abbreviations

ADN: Adiponectin
ALB: Albumin
ALP: Alkaline phosphatase
ANOVA: Analysis of variance
AST: Aspartate aminotransferase
BCS: Body condition score
BUN: Blood urea nitrogen
BW: Body weight
CRE: Creatinine
CRP: C-reactive protein
GPx: Glutathione peroxidase
LALP: Liver alkaline phosphatase
LDH: Lactate dehydrogenase
MDA: Malondialdehyde
MDH: Malate dehydrogenase
M/L: Malate dehydrogenase/lactate dehydrogenase ratio
NEFA: Non-esterified fatty acid
SAA: Serum amyloid A
SE: Standard error
SOD: Superoxide dismutase
TC: Total cholesterol;
TG: Triglyceride
TP: Total protein
